# Cerebral and cognitive correlates of olfactory functioning in older adults at risk of Alzheimer’s disease: preliminary results from the CIMA‐Q Cohort

**DOI:** 10.1002/alz.093979

**Published:** 2025-01-09

**Authors:** Benoît Jobin, Johannes Frasnelli, Benjamin Boller, CIMA‐Q Group

**Affiliations:** ^1^ Université du Québec à Trois‐Rivières, Trois‐Rivières, QC Canada; ^2^ Research Center of the Hôpital du Sacré‐Cœur de Montréal, Montréal, QC Canada; ^3^ Consortium pour l'Identification précoce de la Maladie d'Alzheimer ‐ Québec, Montréal/Québec/Sherbrooke, QC Canada

## Abstract

**Background:**

Olfactory identification is impaired in the early stages of Alzheimer’s disease (AD), including mild cognitive impairment (MCI). This early sensory impairment could result from several factors such as early cognitive or structural cerebral damages caused by the disease. It is however not clear which factors are contributing the most to the olfactory impairment in older people at risk of developing AD. The aim of this study was to explore and determine the cerebral and cognitive correlates of olfactory identification functioning in a cohort of older adults at risk of developing AD.

**Method:**

Here we report preliminary data on 16 participants with subjective cognitive decline (SCD) (mean age: 76.58, SD: 5.53) and 17 patients with MCI (mean age: 79.94, SD: 7.06) from the Consortium for the Early Identification of Alzheimer’s Disease‐Quebec (CIMA‐Q). Olfactory assessment was completed using the University of Pennsylvania Smell Identification Test (UPSIT), while episodic memory was assessed using the Logical Memory subtests of the Wechsler Memory Scale. All participants underwent a clinical examination and neuropsychological assessment. Further, grey matter volume (GMV) of olfactory‐related structures was extracted using CAT12 in a subgroup of 16 participants for whom MRI data were available.

**Result:**

The UPSIT score was significantly correlated with the Logical Memory immediate recall score in the MCI group (r = .53, p = .03) but not in the SCD group (r = ‐.26, p = .33). No significant differences were found regarding olfactory identification between both groups (p = .75). Bilateral middle cingulate cortex (r = .51, p = .04) and caudate (r = .51, p = .04) GMV were correlated to the UPSIT score, while no significant correlation was found for other structures (p > .05).

**Conclusion:**

These preliminary data suggest that short‐term episodic memory, as well as GMV of middle cingulate cortex and caudate are related to olfactory identification in older adults at risk of AD. Future studies to validate these findings will incorporate more participants from the CIMA‐Q cohort.

